# On the mechanism of tumor cell entry of aloe‐emodin, a natural compound endowed with anticancer activity

**DOI:** 10.1002/ijc.33686

**Published:** 2021-06-11

**Authors:** Teresa Pecere, Eleonora Ponterio, Enzo Di Iorio, Modesto Carli, Matteo Fassan, Luisa Santoro, Maicol Bissaro, Giulia Bernabè, Stefano Moro, Ignazio Castagliuolo, Giorgio Palù

**Affiliations:** ^1^ Department of Molecular Medicine University of Padova Padova Italy; ^2^ Hematology Oncology Division, Department of Women's and Children's Health University of Padova Padova Italy; ^3^ Molecular Modeling Section (MMS), Department of Pharmaceutical and Pharmacological Sciences University of Padova Padova Italy; ^4^ Department of Medicine University of Padova Padova Italy; ^5^ Azienda Ospedaliera di Padova Padova Italy

**Keywords:** aloe‐emodin, anticancer activity, somatostatin receptor

## Abstract

Aloe‐emodin (1,8‐dihydroxy‐3‐[hydroxymethyl]‐anthraquinone), AE, is one of the active constituents of a number of plant species used in traditional medicine. We have previously identified, for the first time, AE as a new antitumor agent and shown that its selective in vitro and in vivo killing of neuroblastoma cells was promoted by a cell‐specific drug uptake process. However, the molecular mechanism underlying the cell entry of AE has remained elusive as yet. In this report, we show that AE enters tumor cells via two of the five somatostatin receptors: SSTR2 and SSTR5. This observation was suggested by gene silencing, receptor competition, imaging and molecular modeling experiments. Furthermore, SSTR2 was expressed in all surgical neuroblastoma specimens we analyzed by immunohistochemistry. The above findings have strong implications for the clinical adoption of this natural anthraquinone molecule as an antitumor agent.

**Abbreviations**
Ab: antibodies
AE: aloe‐emodin
BCA: bicinchoninic acid
BSA: bovine serum albumin
DMEM: Dulbecco's Modified Eagle Medium
DMS114: small cell lung carcinoma
DMSO: dimethil sulfoxide
GAPDH: glyceraldehyde 3‐phosphate dehydrogenase
GPCR: G protein‐coupled receptors
HeLa: cervix epithelioid carcinoma cells
IMR32: neuroblastoma cells
KOP: k‐opioid receptor
LoVo: colon adenocarcinoma cells
MRC5: human lung fibroblasts
qPCR: relative quantitative PCR
RNAi: RNA interference
RPMI‐1640: Roswell Park Memorial Institute 1640 Medium
siRNA: short interfering RNA
SST: somatostatin
SST14: cyclic peptide somatostatin‐14
SST‐28: N terminally extended somatostatin‐28
SSTR2: somatostatin receptor 2
SSTR5: somatostatin receptor 5

**What's new?**

Aloe‐emodin is a natural anthraquinone found in different plant species that has been used as an active ingredient in traditional medicine. Among other properties, previous evidence suggests that aloe‐emodin exerts antitumor activities through a cell‐specific drug uptake process. The molecular mechanism underlying the entry of aloe‐emodin into tumour cells however remains to be determined. Here, the authors demonstrate the involvement of somatostatin receptors 2 and 5 (SSTR2 and 5) in aloe‐emodin accumulation and cytotoxicity in tumour cells. The results have strong implications for the potential clinical adoption of this natural anthraquinone molecule as an antitumor agent.

## 1 INTRODUCTION

In the era of combinatorial chemistry and high‐throughput screening of large compound libraries development of natural products has been neglected. However, despite steady progress in the above fields, cancer treatment modalities are unsatisfactory for many solid tumors and there remains an unmet need for new drug discovery.^1^

Aloe‐emodin (AE) is a natural anthraquinone (1,8‐dihydroxy‐3‐[hydroxymethyl]‐anthraquinone) produced by different species of well‐known plants, such as *Aloe* and *Rheum*,^2^ as well as the annelid worms of the *Tomopteris* genus.^3^

AE shows a number of antimicrobial, metabolic, diuretic and immunosuppressive properties including, lastly, anticancer activities.^4^, ^5^, ^6^

We have originally described the selective in vitro and in vivo killing of neuroblastoma cells by AE, without appreciable signs of acute toxicity.^7^ The anticancer activity, in particular, was deemed to result from apoptotic cell death, cellular differentiation and antiangiogenic mechanism.^7^, ^8^, ^9^ In addition, a number of studies showed that AE affected ERK 1/2 pathway in glioma and fibrosarcoma cells^10^, ^11^; it was already reported that different SSTR subtypes 1, 2, 5 and are able to affect cell proliferation of C6 glioma cells in vitro through the activation of the same intracellular pathway (inhibition of ERK1/2 phosphorylation).^12^ We have also demonstrated that the anticancer activity of AE was most likely promoted by a tumor‐cell‐specific drug uptake process.^13^

However, the mechanism for the selective uptake of AE in large cytoplasmic vesicles by tumor cells remains elusive.

Like AE, somatostatin (SST) and derivatives thereof affect a broad range of biological pathways resulting in metabolic, cathartic, cell proliferation, cell survival effects and, notably, antineuroectodermic tumor activity.^14^, ^15^, ^16^, ^17^ In particular, two biologically active forms have been identified in mammals, the cyclic peptide somatostatin‐14 (SST‐14) and the N terminally extended somatostatin‐28 (SST‐28). The biological effects of somatostatin are mediated through a family of G protein‐coupled receptors (GPCR) which comprises five distinct subtypes (SSTR 1‐5).^14^ SSTRS are highly expressed in various cultured tumor cells and primary tumor tissues of neuroectodermal and nonneuroectodermal origin, including neuroendocrine tumors (NET). Generally, each tumor variably expresses more than one somatostatin receptor subtype.^18^, ^19^, ^20^, ^21^

In recent years, considerable interest has been placed on receptor‐targeted cancer therapy since certain receptors are found aberrantly expressed in cancer cells at higher concentrations than in normal cells.^20^, ^21^

Our study, that focuses on the mechanism of AE‐uptake, unveils how this process is mediated by two of the five receptors of somatostatin: SSTR2 and SSRT5.

## 2 MATERIALS AND METHODS

### 2.1 Drugs

Aloe‐Emodin was provided by Angelini S.p.A. and it was dissolved in DMSO stock solution of 200 mM. Somatostatin14 was purchased from Sigma‐Aldrich (Sigma‐Aldrich, Milan, Italy). All drugs were stored at −20°C.

### 2.2 Cell culture

Neuroblastoma cells, IMR32 (RRID:CVCL_0346), human lung fibroblasts, MRC5 (RRID:CVCL_0440), cervix epithelioid carcinoma cells, HeLa (RRID:CVCL_0030) and colon adenocarcinoma cells, LoVo (RRID:CVCL_4Y03) were purchased from ATCC (American Type Culture Collection, Manassas, VA), small cell lung carcinoma, DMS114 (RRID:CVCL_1174) were from the National Cancer Institute (NCI, New York, NY). MRC5, HeLa and LoVo cells were grown in DMEM medium, DMS114 and IMR32 were grown in RPMI‐1640 medium (Lifetechnologies Gibco, Milan, Italy). Culture medium was supplemented with 10% heat‐inactivated fetal bovine serum (Lifetechnologies Gibco, Milan, Italy), 100 units/mL penicillin and 100 μg/mL streptomycin (Lifetechnologies Gibco, Milan, Italy). All cell lines were grown at 37°C with 5% CO_2_ humidified atmosphere. All experiments were performed with mycoplasma‐free cells.

All human cell lines have been authenticated using STR profiling within the last 3 years.

### 2.3 Cell viability assay

Cells were seeded in 96‐well plates in regular growth medium. After 24 hours of incubation, medium was discarded and replaced by 100 μL of regular growth medium supplemented with different concentrations of AE for 72 hours. Then, cells were washed twice with PBS, and cell viability was evaluated using Cell Proliferation Kit I (Cell Proliferation Kit I, Roche Diagnostics GmbH, Mannheim, Germany) according to the manufacturer's instructions. All assays included cell treatment with the vehicle (DMSO) dissolved in growth medium at 0.05% concentration. All experiments were conducted at least in triplicate.

### 2.4 Analyses of RNA expression

Real‐time PCR analyses were carried out to quantify expression of the selected *sstrs* genes in IMR32, DMS114 and MRC5 cells. Total RNA was isolated using the Qiagen RNeasy Mini Kit in accordance with the manufacturer's instructions. cDNA synthesis were performed using GeneAmp RNA PCR Core Kit (Roche, New Jersey). The expression levels of *sstrs* mRNA were evaluated by qualitative RT‐PCR using the specific primers.

Relative quantitative PCR (qPCR) was carried out in 25 μL final volume utilizing 3 μL of cDNA, 0.2 μM of each specific primers, using SYBRgreen reagents according to the manufacturer's instructions. PCR reactions comprised 40 cycles after an initial denaturation step (95°C, 10 minutes) according to the parameters: denaturation at 95°C, 15 seconds and annealing/extension at 60°C, 1 minute, in an ABI PRISM 7700 Sequence Detection system. To normalize the RNA amount of the extracted samples, real‐time PCR analysis of the human GAPDH cDNA was carried out. Moreover, we optimized the method for relative quantification with sequence‐specific DNA probes for *sstr2* and *sstr5* genes TaqMan reagents using kit TaqMan Gene Expression Assays according to the manufacturer's instructions (Applied Biosystem, Milan, Italy). The relative quantification of a target template in the samples was evaluated using the 2^−ΔΔCT^ method. All experiments were conducted in triplicate (details on primers are reported in Appendix S1).

### 2.5 Western‐blot analyses

IMR32 and MRC5 cells were seeded and incubated with or without AE (50 μM). The collected cells were solubilized on ice in RIPA lysis buffer (SIGMA, Milan, Italy) supplemented with complete protease inhibitor cocktail tablet (Promega, Milan, Italy). Followed by centrifugation at 14 000 rpm for 5 minutes at 4°C. The protein concentration was determined by BCA assay (Pierce, Thermo Fisher), using BSA as a standard according to the manufacturer's instructions. Equal amount of total proteins (40 μg) were subject to Western blot analyses. The membranes were blocked with 5% w/v nonfat dry milk in PBS with 0.1% Tween‐20 and blots were probed with specific antibodies (Ab) to SSTR2, SSTR5 and GAPDH (Sigma, Milan, Italy). Blots were visualized with a peroxidase‐conjugated anti‐rabbit IgG secondary antibody (Santa Cruz, CA) and developed with enhanced chemiluminescence reagents (Pierce Thermo Fisher, Milan, Italy). Pixels intensity of the visualized bands was determined by using Image J software (W. S. Rasband, NIH, Bethesda, MD [http://rsb.info.nih.gov/ij/]). All experiments were conducted in triplicate.

### 2.6 Molecular modeling

Since no crystallographic structure of the somatostatin receptor remains available, two homology models were constructed, using as template the recently published crystal structure of the k‐opioid receptor (KOP) (PDB ID: 6B73), given the high sequence similarity with both SSTRs subtypes considered in our study. The quality of models was evaluated using the SWISS‐MODEL workspace (details are reported on Appendix S1).

Three‐dimensional structures of the ligands under investigation were built and correctly prepared taking advantage of the MOE suite, following the protocol described in Appendix S1.

GOLD docking tool was selected as a conformational search program and PLP as a scoring function.^22^ In total 20 docking runs were performed for each somatostatin receptor, searching in a sphere of 15 Å radius. Along with AE, the compound under investigation, docking simulations were conducted also for ligands L‐779976 and L‐817818, the references nonpeptidic potent and selective agonists respectively of SSTR2 and SSTR5.

### 2.7 Cell viability modulation by *sstr*‐2 and *sstr*‐5 antibodies and SST14

IMR32 cells were seeded as described above. After 24 hours of incubation the medium was discarded and replaced by 100 μL of medium supplemented with AE (50 μM), SST14 (10 μM), 1:500 SSTR2 Ab, SSTR5 Ab (Santa Cruz, CA), or SST14 with AE (50 μM), or SSTR2 Ab and SSTR5 Ab together with AE (50 μM). Then the cells were evaluated for viability, as mentioned before.

### 2.8 Confocal microscopy analyses

IMR32 and MRC5 cells were seeded on microscope coverslips in 12‐well plates and cultured with drug‐free medium 24 hours before treatment. Cells were treated with the compounds and observed in vivo. AE was added at the final concentration of 50 μM. IMR32 and MRC5 cells were also pretreated with SST14 at 10 μM for 20 minutes and with SSTR5 and SSTR2 Abs (Santa Cruz, CA) – 1:100 – for 20 minutes.

AE fluorescence was collected during the incorporation and was evaluated as a ratio between the basal fluorescence of the cells and the maximum value of fluorescence reached in 3 minutes. Fluorescence was monitored with excitation at 488 nm and emission at 500‐550 nm. Confocal images were obtained using A1Rsi + Laser Scan Confocal microscope (Nikon Instruments Inc., Melville, NY) with a detection system and a ×20 objective. Illumination intensity was 5% laser. Data are presented as change in fluorescence relative to the initial fluorescent value for each individual cell. Images were captured every 5 seconds. Analysis of fluorescence intensity was performed after image acquisition using NIS‐Elements Advanced research (Nikon Instruments Inc.). AE fluorescence (*F*) was expressed as a percentage collected during the incorporation and was evaluated (Δ*F*%) as medium of 30 regions of interest (ROIs): [(maximum value of fluorescence − basal fluorescence)/ basal fluorescence] × 100.

### 2.9 siRNA‐mediated *sstr*5 gene knockdown

Gene‐specific pooled siRNA trilencer targeting human *sstr5* and a scrambled negative control duplex were purchased from Origene (OriGene Technologies, Rockville, MD). IMR32 cells were transfected with 20 nM aliquots of human sstr5 siRNA and control siRNA by using Lipofectamine RNAiMAX (Invitrogen, Thermo Fisher Scientific, Waltham, MA) following the manufacturer's instructions. Twenty‐four hours before transfection 30 000 cells were plated in 500 μL of growth medium without antibiotics for 24 hours. Then the cells were transfected with RNAi duplex‐Lipofectamine RNAiMAX complexes, final RNA concentration of 20 nM, for 20 minutes at room temperature.

Cells were incubated at 37°C in a CO_2_ incubator until assayed for gene knockdown. Gene silencing was stable for 72 hours. After 48 hours cells were treated with AE (50 μM). Twenty‐four hours after AE treatment cells were harvested and prepared for quantitative proliferation assay, as previously described. Nontargeting siRNA transfected cells were used as a negative control.

### 2.10 Immunostaining

Paraffin embedded surgical neuroblastoma specimens (n = 9) were retrieved from the archives of the Surgical Pathology Unit of the University Hospital of Padua. Immunostaining was automatically performed using the Bond Polymer Refine Detection kit (Leica Biosystems, Newcastle Upon Tyne, UK) in the BOND‐MAX system (Leica Biosystems) on 3‐4 μm thick sections with the primary antibodies for SSTR2 (antisomatostatin receptor 2 antibody [UMB1]; Abcam, Cambridge, UK) and SSTR5 (antisomatostatin receptor 5 antibody [UMB4]; Abcam, Cambridge, UK).

The staining intensity thresholds were set as follows: strong, (3+); moderate, (2+); weak, (1+). The total number of cells for each staining intensity was calculated and a modified H‐score was calculated for each tumor with the following formula: (1 × [% cells 1+] + 2 × [% cells 2+] + 3 × [% cells 3+])/100. The scores lay on a continuous scale from 0 to 3.^23^

### 2.11 Statistical analyses

Statistical analyses were performed with Student's unpaired *t*‐test with *P < .05 and **P < .01.

## 3 RESULTS AND DISCUSSION

Following our original report^7^ on AE activity against neuroectodermal tumor cells, a number of publications have shown AE activity also on tumor cells of different origins,^4^, ^5^, ^6^, ^7^, ^8^, ^9^, ^10^, ^11^ a result that seems to contradict, at least in part, our previous report of AE selectivity. In order to understand these apparently conflicting observations, we approached the search for an AE cellular specific receptor. We have already demonstrated that AE is incorporated at high concentrations in sensitive cells. In neuroblastoma cells AE was incorporated with an intracellular concentration of 570‐fold over the cellular background. The fluorometric signal in MRC5 and HeLa cells did not allow to appreciate a relative increase in drug uptake.^13^ In order to evaluate a reduction of AE uptake in the sensitive cells we assayed different ligand and/or antibodies of a number of neuroectodermal tumor cell receptors, such as nerve growth factor (NGF), acetylcholine, somatostatin (SST14), clonidine, adrenaline and noradrenaline. We evaluated the relative amount of AE uptake by different analyses: two‐photon excitation microscopy (TPE) and flow cytometry (data not shown). This screening showed that sst14, sstr2 and 5 antibodies significantly reduced cellular AE uptake. Hence the role of these receptors seems quite relevant in the above process although we cannot exclude the role played by other receptors such opioid and G‐coupled receptors that are abundantly present in IMR32 cells.^24^ Considering the similarity of the pleiotropic effects of both somatostatin peptides and AE, we investigated if and how somatostatin receptors were expressed on representatives of AE‐sensitive and AE‐no sensitive cell lines.

### 3.1 Cell viability

**FIGURE 1:**
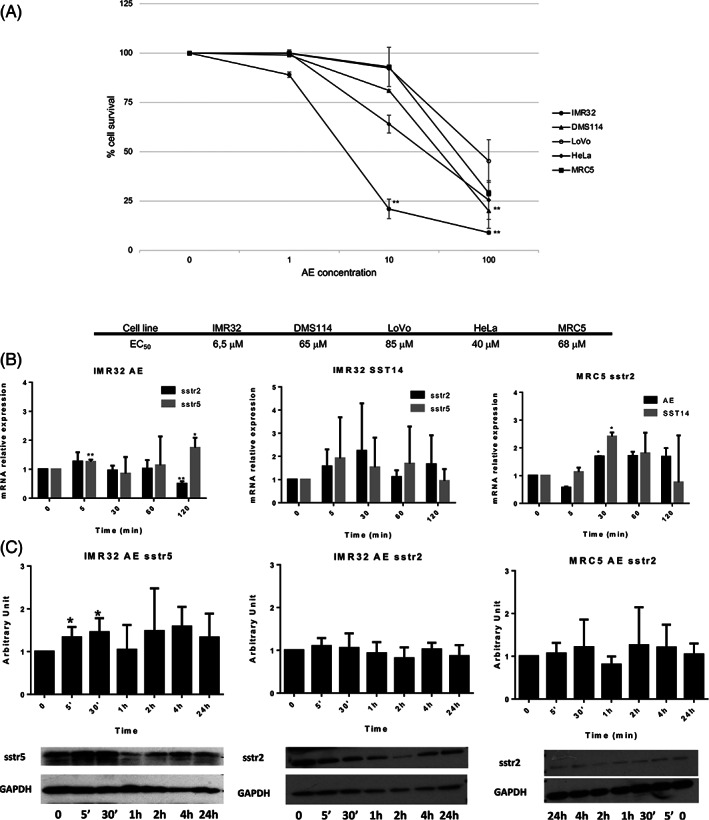
Panel A – Cell viability assay. Cells were treated with different concentrations of AE (1‐10‐100 μM) for 72 hours. The viability was compared to the untreated cells. The plate absorbance (ABS) was measured in a spectrophotometer, at 620 nm wavelength. Cell survival was expressed as a percentage calculated as follow [(ABS AE treated cells − ABS Background)/(ABS Vehicle treated cells ‐ ABS Background)] × 100 with MTT analysis. Panel B – *Sstr2* and *sstr5* mRNA gene expression. IMR32 and MRC5 cells were exposed to AE (50 μM) and SST14 (10 μM) for different time points (5, 30, 60 and 120 minutes). *Sstr5* gene was not expressed in MRC5 cells. Panel C – Western‐blot analyses: SSTR5 and SSTR2 protein expression after AE treatment (50 μM) in AE‐sensitive cells (IMR32) and in AE nonsensitive cells (MRC5). Densitometric analysis of data from representative of three experiments was presented as fold increase relative control

When AE cytotoxicity was evaluated on a panel of cell lines of different embryonic origin, a more significant cytotoxic activity was observed on IMR32 cells vis a vis DMS114, HeLa, MRC5 and LoVo cells (Figure 1A). Noteworthy IMR32 and DMS114 cells are of neuroectodermal origin, whereas HeLa, MRC5 and LoVo cells are of mesodermal and ectodermal origin.

Of the two cell lines of neuroectodermal origin, IMR32 expressed SSTR2 and 5, whereas DMS114 expressed no somatostatin receptor. MRC5, taken as a cell line representative of nonneuroectodermal origin, expressed mRNA for SSTR1 and 2 (data not shown).

### 3.2 RNA expression and western‐blot analyses

The *sstr5* gene expression, but not *sstr2* gene expression, was significantly up‐regulated in IMR32 cells by exposure to AE. At variance, *sstr2* and *sstr5* genes were both up‐regulated by somatostatin‐14, but not at a significant level (Figure 1B). The *sstr5* gene was not expressed in MRC5 cells but *sstr2* gene was significantly up‐regulated both by AE and SST14 (Figure 1B).

**FIGURE 2:**
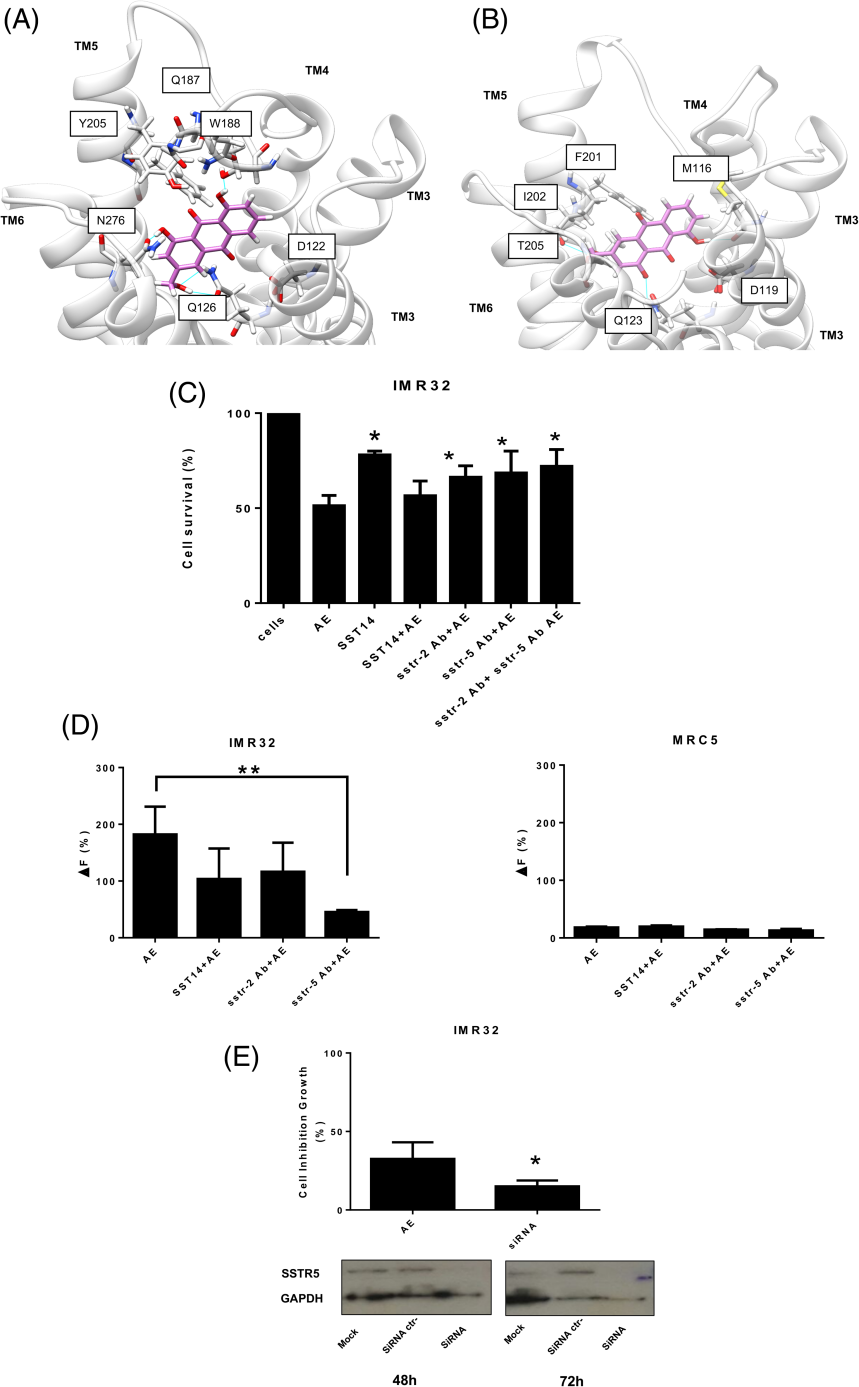
Panel A – Molecular docking predicted binding modes for Aloe‐emodin in complex with the SSTR2 and the SSTR5 homology model (Panel B); residues involved in molecular recognition are labeled while hydrogen bond between ligands and the receptor are depicted by the blue lines. Panel C – IMR32 cells viability modulation. After 24 hours of treatment with AE, SST14 or co‐treatment with AE and SST14 or Abs the cells were evaluated for viability. Panel D – Confocal microscopy analyses. IMR32 and MRC5 cells were pretreated for 20 minutes with SSTR2, SSTR5 Abs or SST14 and relative fluorescence amount in the cells was evaluated in 3 minutes from AE administration, as mentioned in section “Materials and Methods.” Panel E – SiRNA knockdown. IMR32 cells were transfected with siRNA for somatostatin receptor 5. Western blot analysis for SSTR5 protein expression shown that gene silencing occurred 48 hours after siRNA transfection and was stable for 72 hours. IMR32 transfected cells were treated with AE (50 μM) for 24 hours and evaluated for the proliferation inhibition [Color figure can be viewed at wileyonlinelibrary.com]

Western blot analyses confirmed the above findings (Figure 1C). It is reasonable to conceive that SSTR5 must play an epistatic role over SSTR2 in IMR32 cells. The latter receptor, in turn, being the sole receptor expressed in MRC5 cells, is the only one undergoing positive feedback by AE. In order to find a plausible role for these receptors in AE‐sensitive cells, molecular docking simulations were exploited using the two SSTR subtypes that were significantly expressed and up‐regulated. Since AE is a molecule considerably smaller than the endogenous agonist SST or the reference nonpeptide compounds, the explorable space by the ligand within the orthosteric binding site is large enough to increase the variability of docking predicted poses. The most reasonable binding modes of AE, respectively, within SSTR2 (Figure 2A) and SSTR5 binding sites (Figure 2B) are shown. AE interacts mainly with residues of the transmembrane segments TM3, TM5, TM6 and with the extracellular loop EL2, a portion of the binding site very similar to the one described for the reference agonist compound (more details are reported on Appendix S1).

### 3.3 Cell viability modulation

In order to obtain a functional validation of the gene expression experiments and molecular dynamic prediction, cell viability modulation was studied using the natural ligand SST14 as well as antibodies against SSTR2 and SSTR5. As explained in Figure 2C, SST14 was less cytocidal for IM32 cells than AE alone or in combination with AE. It is, therefore, conceivable that both molecules competed for the same receptors affecting AE‐sensitivity in a nonsynergistic nonadditive mode. A significant increasing cell survival was observed in AE and SSTR2 and/or SSTR5 antibodies co‐treated cells indicating the relevant role of these receptors for AE activity. It was previously described that AE strongly inhibited ERK1/2 signaling pathway affecting proliferative potential of C6 astrocytoma and fibrosarcoma cells.^10^, ^11^ In the experimental model of C6 glioma cells, cytostatic effects of somatostatin were mediated by the SSTR2, and SSTR5,^12^ the likely carriers of AE. One could therefore assume that the ERK 1/2 pathway could also be targeted by AE in our cell line.

### 3.4 Confocal microscopy analyses

Being an autofluorescent molecule (*λ*_ecc_ at 410 nm and *λ*_em_ at 610 nm) AE accumulation was investigated in sensitive and nonsensitive cells after treatment with antibodies and the somatostatin natural ligand, SST14. Fluorescence was significantly down‐regulated in IMR32 cells after incubation with SSTR5 *Ab*; only a minor difference in AE accumulation was observed after SST14 or SSTR2 *Ab* pretreatment (Figure 2D). Besides, neuroblastoma cells exhibited no significant variation in survival after 24 hours of co‐exposure to AE and SST14 (Figure 2C). These findings would prompt us to suggest that AE, although strongly competing for the same receptor as SST14 is, with respect to this ligand, the primary cytotoxic agent having a diversified mechanism of action that also includes DNA binding, as previously shown by us.^13^ AE accumulation fluorescence was not evident in MRC5 cells treated or not treated with SSTR2 and SSTR5 antibodies or SST14 (Figure 2D).

### 3.5 Gene knockdown

IMR32 cells, as a representative cell line highly expressing SSTR5, were also transfected with *sstr5* siRNA. After gene silencing cells were significantly less sensitive to AE growth inhibition (Figure 2E).

**FIGURE 3:**
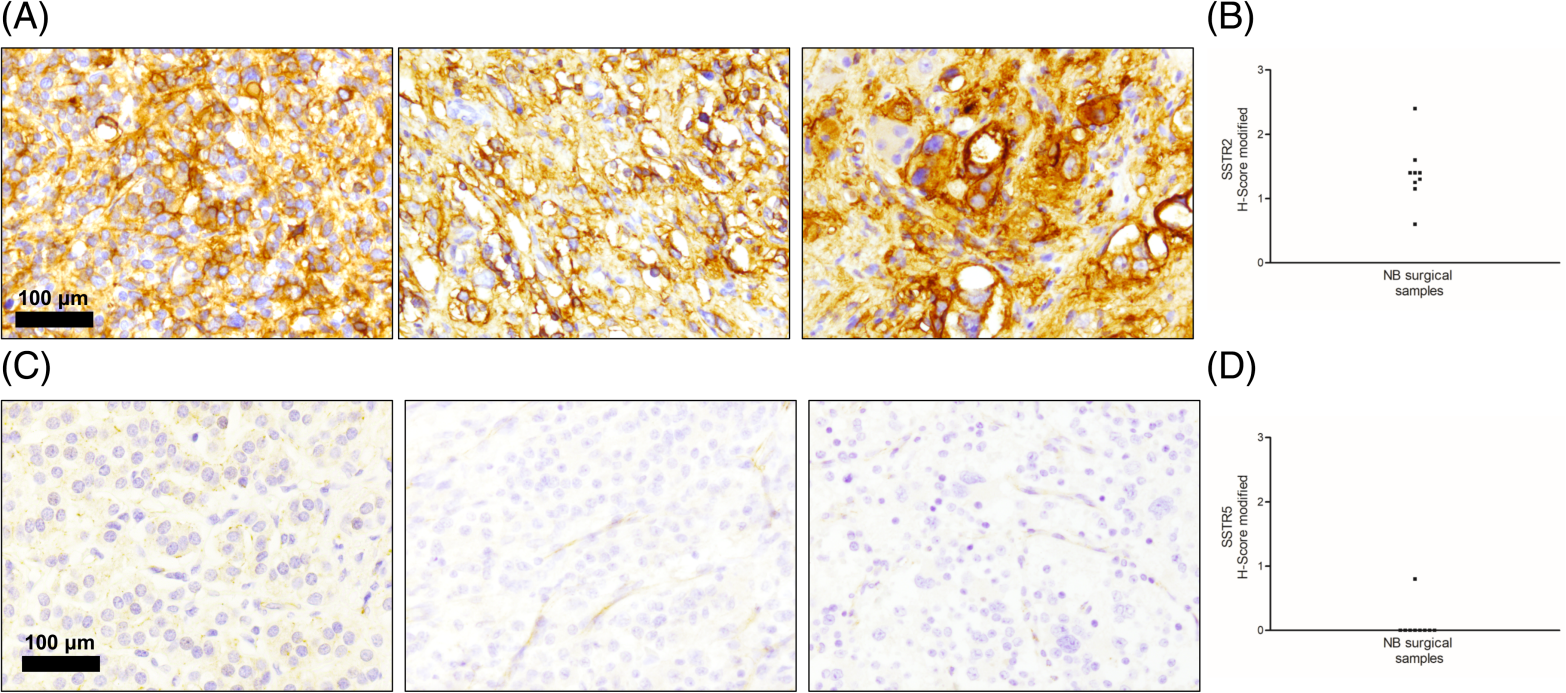
Panel A – Immunohistochemistry for SSTR2. Representative immunohistochemistry for SSTR2 in archival neuroblastoma surgical specimens showing strong immunoreactivity. Scale bar, 100 μm. Panel B – SSTR2 H‐score in neuroblastoma surgical specimens. Panel C – Immunohistochemistry for SSTR5. Representative immunohistochemistry for SSTR5 in archival neuroblastoma surgical specimens showing faint (Case 1) or absent (Cases 2 and 3). Scale bar, 100 μm. Panel D – SSTR5 H‐score in neuroblastoma surgical specimens [Color figure can be viewed at wileyonlinelibrary.com]

Since we proposed neuroblastoma neoplasia as possible targets for AE treatment, standing the relatively high sensitivity of neuroblastoma cell lines to the drug,^7^, ^13^ the in vivo function of SSTR^18^, ^21^, ^25^ was the most intuitive one to explore. To this end we evaluated SSTR2 and SSTR5 immunoreactivity in neuroblastoma's surgical specimens. While SSTR5 immunoreactivity was barely detectable in one out of the nine tumor specimens evaluated, a moderate to strong immunoreactivity for SSTR2 was detectable in all neuroblastoma sections examined (Figure 3).

## 4 DISCUSSION

The original contribution of this article is the demonstration that somatostatin receptors 2 and 5 are likely responsible for AE accumulation and cytotoxicity.

Our results of gene amplification, western blotting, and imaging techniques, indicate that SSTRs were expressed in a different way in neuroectodermal cells and in AE‐sensitive and nonsensitive cells and that AE has the biological and molecular characteristic to be recognized by SSTR2 and SSTR5. In particular, these receptors are induced after AE treatment. Moreover, a different pattern of SSTR2 and SSTR5 expression is shown in AE‐sensitive and nonsensitive cells, that correlates with the differential cytotoxic potential of AE, receptor knockdown and competition experiment. These data are corroborated by our preliminary molecular modeling studies showing how AE can be recognized by SSTR2 and SSTR5 receptors establishing, in the orthosteric binding sites, a network of stabilizing interactions (Figure 2 and Appendix S1). In conclusion, we considered these findings as the molecular and biological explanation of the AE selectivity we originally reported for neuroectodermal tumors. Moreover, our observations on the receptor‐mediated mode of action of the anthraquinone compound, combined to the strong and widespread expression of SSTR2 in human neuroblastoma specimens and prompt to a further development of AE as a personalized antitumor agent and to explore its potential use for other biological application mediated by somatostatin receptors.

## CONFLICT OF INTEREST

The authors declare no conflicts of interest.

## Supporting information

**Appendix S1** Supporting Information.

## Data Availability

The data that support the findings of our study are available from the corresponding author upon reasonable request.
