# Supplementary material for: On the mechanism of tumor cell entry of aloe‐emodin, a natural compound endowed with anticancer activity
Source: Int J Cancer. 2021 Jun 11;149(5):1129–36. doi: 10.1002/ijc.33686 (PMC8361998; doi:10.1002/ijc.33686)
Supplement: Supplementary file 1 — Appendix S1 Supporting Information. [file IJC-149-1129-s001.pdf]

ON THE MECHANISM OF TUMOR CELL ENTRY OF ALOE-EMODIN, A NATURAL COMPOUND ENDOWED WITH ANTICANCER ACTIVITY

Teresa Pecere[1], Eleonora Ponterio[1], Enzo Di Iorio[1], Modesto Carli[2], Matteo Fassan[4], Luisa Santoro[5], Maicol Bissaro[3], Giulia Bernabè[1], Stefano Moro[3], Ignazio Castagliuolo[1], Giorgio Palù[1*]

Table of contents:

1. Supplementary Materials and Methods

2. Supplementary Results

3. Supplementary Discussion

4. Supplementary References

1. Materials and methods

Molecular modeling

1.1. Preparation and validation of the Homology Models of the SSTR2 and the SSTR5

Homology modeling is based on the assumption that protein with a similar sequence also shares a conserved three-dimensional structure; this is particularly true for the family of GPCRs, transmembrane receptors characterized by conserved TM boundaries and overall topology.

As template for the construction of the model, the recent published crystal structure of the k-opioid receptor (KOP) was chosen (PDB ID: 6B73), given the high sequence similarity with both somatostatin receptor isoforms considered in this study (SSTR2/KOP shows sequence identity of ~ 40% and SSTR5/KOP shows sequence identity of ~ 42%).[1]

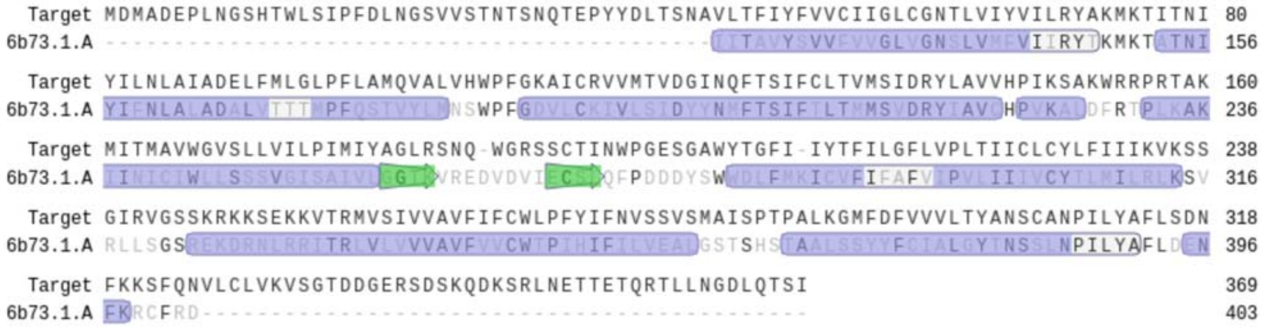

Supplementary figure 1 Sequence alignment of SSTR2 and KOP. In purple are highlighted the residues belonging to seven TM helices while in bold are identified the conserved residues.

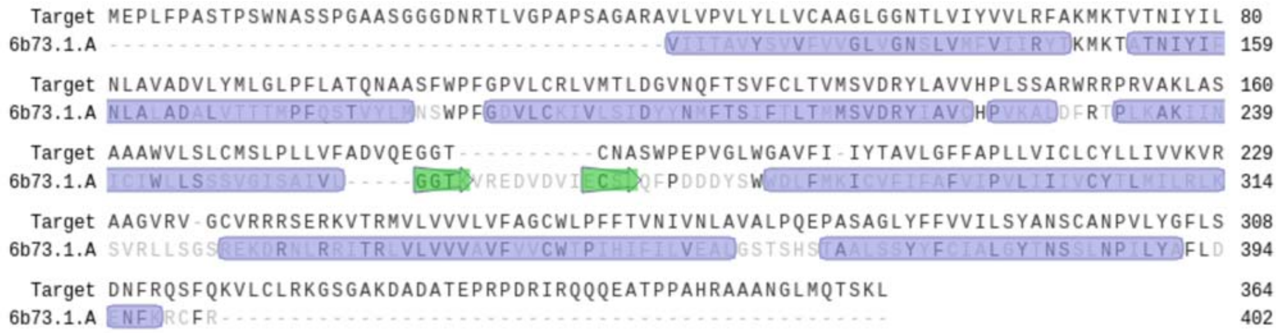

***Supplementary figure 2** Sequence alignment of SSTR5 and KOP. In purple are highlighted the residues belonging to seven TM helices while in bold are identified the conserved residues.*

The numbering of the amino acids follows the arbitrary scheme by Ballesteros and Weinstein.[2] The amino acids sequences belonging to the seven TM helices of both SSTRs subtypes were aligned with those of the template structure, as highlighted with purple color in supplementary figures 1 and 2. The alignment was guided by the presence of highly conserved amino acids residues, such as the DRY motif (Asp3.49, Arg3.50, Tyr 3.51) and the NPxxY motif (Asn7.49, Pro7.50, Tyr 7.53). The same boundaries were applied to the TM helices of KOP crystal structure, the coordinates of which were then used to construct the seven TM architecture of both SSTRs subtypes. The loop domains were constructed using the loop search method as implemented in MOE suite, based on the structure of compatible loop fragments found in the protein data bank (PDB).[3,4] Side chains were modelled using a library of rotamers generated by systematic clustering of the PDB data. Side chains belonging to residues whose backbone coordinate were copied from the KOP template were modeled first, while side chains of residues belonging to loops were modeled secondly. Outgaps and their side chains were modeled last. The cysteine residues involved in the disulfide bridge between TM3 and EL2 in the KOP receptor were selected to be constrained with the correspondent cysteine found in the SSTRs. In particular, Cys3.25 (TM3) and Cys210 (EL2) of the KOP receptor were constrained, respectively, with Cys3.25 (TM3) and Cys193 (EL2) of the SSTR2 and with Cys3.25 (TM3) and Cys186 (EL2) of the SSTR5. The presence of the disulfide bridges in the models were manually checked at the end of the homology

modeling procedure. Once the heavy atoms of SSTRs structures have been modeled, the hydrogen atoms were added and minimized in MOE using the AMBER12 force field.[5] The minimizations were carried out by the 1000 steps of steepest descent followed by conjugate gradient minimization until the RMS gradient of the potential energy was less than 0.1 kcal mol^{-1} $Å^{-1}$. Protonate 3D tool, part of the MOE suite, was exploited for the assignment of the correct protonation state of every ionizable residue. [6]

The SWISS-MODEL workspace (https://swissmodel.expasy.org/) was exploited to validate the quality of the models.[7,8] In particular, local estimates of the model quality based on the QMEAN scoring function are shown as a per-residue plot, while the global quality is calculated in relation to a set of high-resolution PDB structures (Z-score).

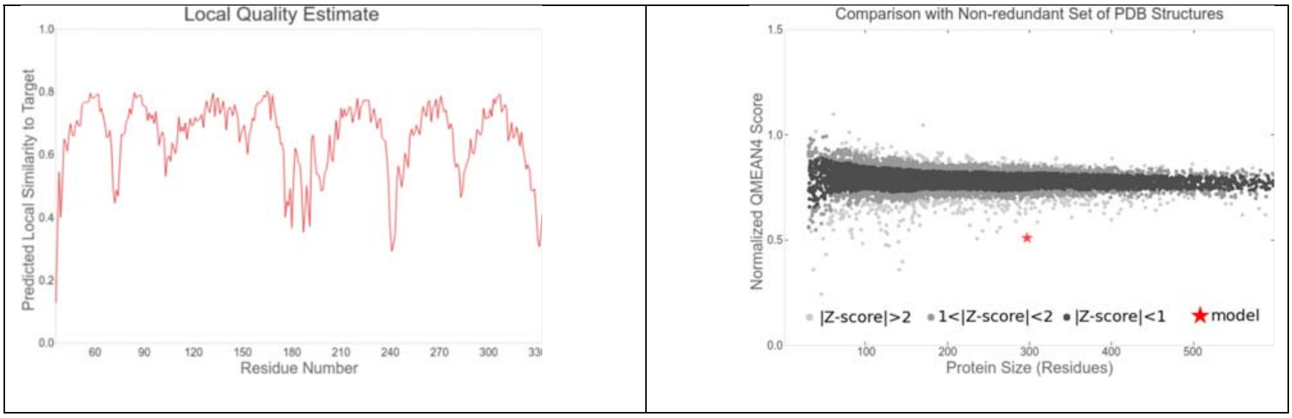

Supplementary *figure 3* *Local and global quality assessment of the SSTR2 model. In the left panel is plotted a score associated to each residue of the model (reported on the x-axis) reflecting the expected similarity to the native structure (y-axis). Residues belonging to the seven TM helices are characterized by high scores (good model quality) while the residues belonging to the loop segments show lower quality (score below 0.6). In the right panel is reported a plot showing the quality of the model, as normalised QMEAN score (y-axis), in comparison to the scores obtained analysing PDB structures. Higher values indicate that the model is of comparable quality to experimental structures of similar size.*

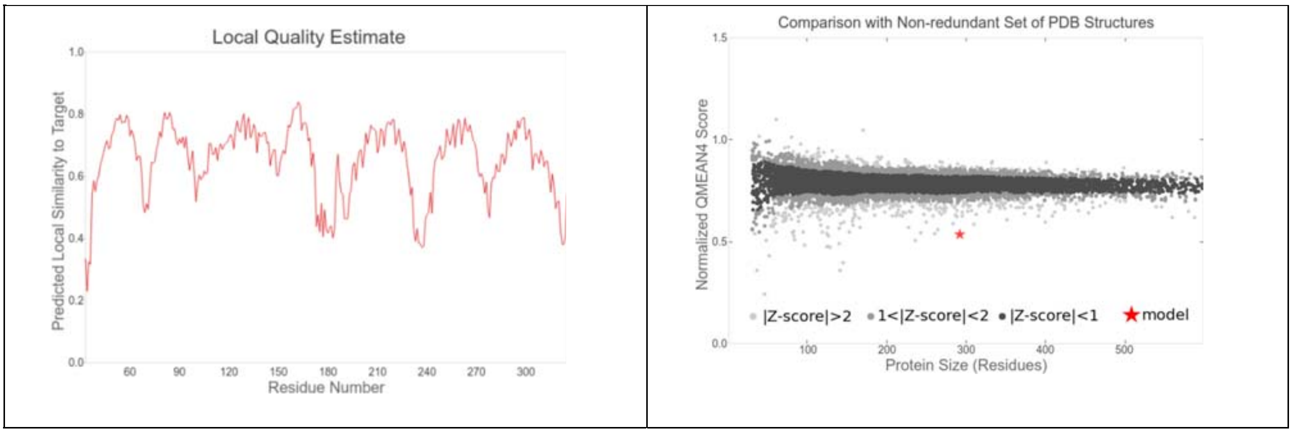

Supplementary *figure 4* *Local and global quality assessment of the SSTR5 model. In the left panel is plotted a score associated to each residue of the model (reported on the x-axis) reflecting the expected similarity to the native structure (y-axis). Residues belonging to the seven TM helices are characterized by high scores (good model quality) while the residues belonging to the loop segments show lower quality (score below 0.6). In the right panel is reported a plot showing the quality of the model, as normalised QMEAN score (y-axis), in comparison to the scores obtained analysing PDB structures. Higher values indicate that the model is of comparable quality to experimental structures of similar size.*

1.2. Molecular Docking

Three-dimensional structures of ligands under investigation were built taking advantage of the MOE-Builder tool and ionization states were predicted using the MOE-Protonate-3D tool.[3,6] Ligands structures were subjected to MMFF94× energy minimization until the root mean square (RMS) gradient fell below 0.05 kcal mol^{-1} $Å^{-1}$. GOLD docking tool was selected as a conformational search program and PLP as a scoring

function.[9] In total 20 docking runs were performed for each somatostatin receptor, searching in a sphere of 15 Å radius. Since no crystallographic structure of the somatostatin receptor is still available, we assumed that the binding site was the same as the opioid receptor used as a template in the Homology Modelling procedure. Thus, the coordinates to define the center of the binding site for docking calculation have been predicted using ligand CVV center of mass (PDB ID 6B73). Along with the compound under investigation, docking simulations were conducted also for ligands L-779,976 and L-817,818, the references non-peptidic potent and selective agonists respectively of SSTR2 and SSTR5.[10] After computing atomic partial charges both of ligands poses, using the PM3/ESP method, and receptor, using Amber14 force field, electrostatic and van der Waals contributions to the binding energy were calculated with MOE.[11]

1.3. Interaction Energy Fingerprints (IEFs)

Individual electrostatic and hydrophobic interactions, hereinafter identified as IE_{ele} and IE_{hyd}, respectively, were computed between ligand poses and each protein residue involved in binding.[12] Both these contributions were computed using MOE and, in particular, IE_{ele} were calculated as nonbonded electrostatic interactions energy term of the force field, so they are expressed in kcal/mol. Instead, IE_{hyd} were computed as contact hydrophobic surfaces and are associated with an adimensional score (the higher the better). The data obtained by this analysis were reported in a graphic, called Interaction Energy Fingerprints (IEFs), representing residues (x-axis) in the form of equally high rectangles rendered according to a colorimetric scale. As regards IE_{ele}, colors from blue to red represent energy values ranging from negative to positive values; for IE_{hyd}, colors from white to dark green depict scores going from 0 to positive values. More precisely, we retrieved the coordinates of the center of mass of ligand CVV in the structure of the KOP (PDB ID 6B73), used as a template for the homology model. Only residues within 15 Å from this point were retained as belonging to the binding site and plotted in the IEFs.

Analyses of RNA Expression

Primers	Sequence
β-actin	5'-ATG TCA AAC GTG CGA GTG TC-3'<br>5'-TCT CTG CAG TGC TTC TCC AA-3'
SSTR1	5'- GCCTTATCTTTCCCGAGTGAACACC-3'<br>5'- GGACCTGAGTGAACCATTCGCAAA -3'
SSTR2	5'- ACTCCCTGCACCATCATCGTT -3'<br>5'- TGGAATTTCATGTCTGCATTGCGTA -3'
SSTR3	5'- GGCGTTAGTCTCAGAATCTCGTG -3'<br>5'- TGCACCTCAGTTTCGTCATATGGA -3'
SSTR4	5'- CATGGTCGCTATCCAGTGCA -3'<br>5'- GTGAGACAGAAGACGCTGGTGAACAT -3'
SSTR5	5'- TCTGCTAAGTTAAAGACACCCGAAA -3'<br>5'- CAGCCACACTCCCCTTACACC -3'

Table 1A

Primers	Sequence
GAPDH	5'-CCA CTC CTC CAC CTT TGA CG-3'<br>5'-CAT GAG GTC CAC CAC CCT GT-3'
SSTR2	5'-TAT GTC ATC CTC CGC TAT GCC-3'<br>5'-CAG AGC CAC CTG CAT AGC C-3'
SSTR5	5'-CCC TTC TTC ACC GTC AAC AT-3'<br>5'-CTT GCG GAG GCA CAG AAC CTT-3'

Table 1B

Panel 1A - Primers, performing qualitative RT-PCR. All quantifications were normalized to β-actin expression.

Panel 1B - Primers, performing quantitative RT-PCR. All quantifications were normalized to GAPDH expression.

2. Results

A structure-based molecular modeling study was conducted to rationalize and investigate the ability of compound Aloe Emodin (AE) to recognize the human somatostatin receptors (SSTRs), focusing the attention toward the SSTRs subtypes 2 and 5. For each receptor subtype the binding mode of a reference compound, a potent and selective non-peptidic agonist, was also evaluated in a comparative fashion.

Structure	Name	SSTR2 K_i (nM)	SSTR5 K_i (nM)
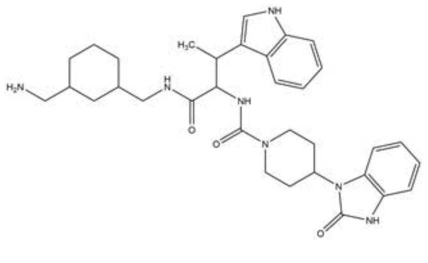	L-779,976	0.05	2009
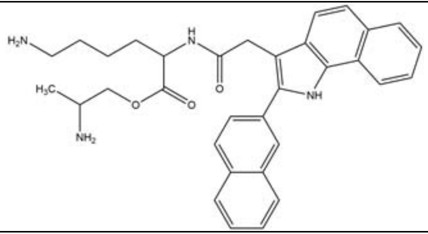	L-817,818	52	0.4

2.1. Docking studies on SSTR2

In supplementary figure 5 (panel A) is reported the energetically more stable binding mode for the reference compound L-779,976 inside the SSTR2 canonical orthosteric binding site, predicted by molecular docking. In the proposed recognition mode, the ligand interacts mainly with residues of the transmembrane segments TM3, TM5, TM6 and with the extracellular loop EL2. The electrostatic and hydrophobic contributions to binding are quantitatively depicted in supplementary figure 6 in a per-residue analysis, through a heat maps representation. A possible binding mode of the anthraquinone compound, Aloe Emodin (AE), was then investigated using the same docking protocol. Since AE is a molecule considerably smaller than the endogenous agonist somatostatin or the reference non-peptidic compound L-779,976, the explorable space by the ligand within the orthosteric binding site is large enough to increase the variability of docking predicted poses. In supplementary figure 5 (panel B) is shown one of the most reasonable binding mode of AE, in which the ligand occupies a portion of the binding site very similar to the one described above for the reference compound. Despite the geometrical similarity, the heat maps reported in supplementary figure 6 show a substantial energetic difference between the electrostatic and hydrophobic contributions to binding mediated by AE, if compared with the reference compound. In conclusion, even if the docking procedure has suggested a possible modality of recognition between AE and the SSTR2, it is reasonable to hypothesize a lower binding stability of AE-SSTR2 complex, with respect to L-779,976.

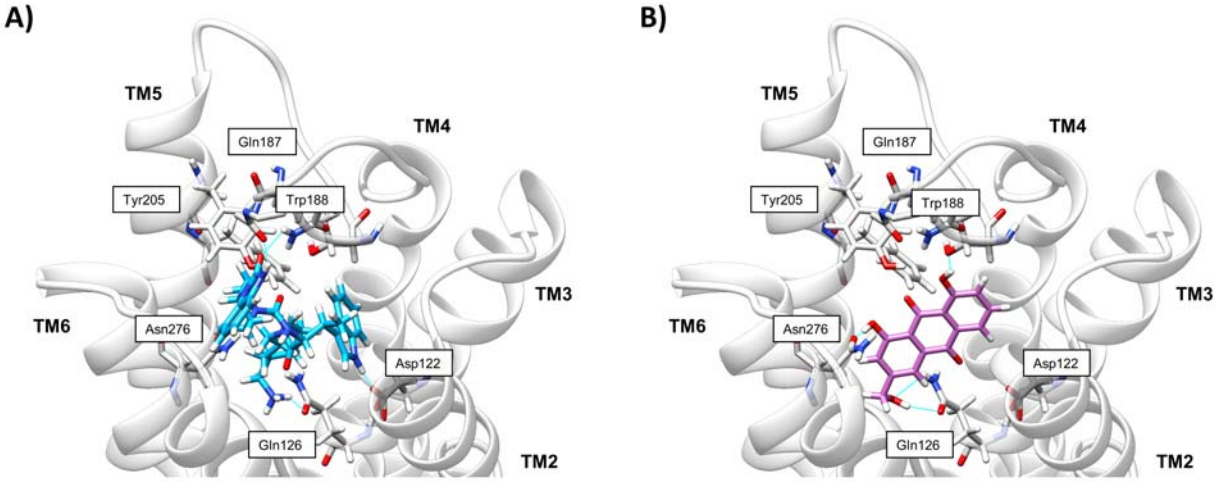

Supplementary figure 5 *Molecular docking predicted binding mode for the reference compound L-779,976 (A) and Aloe Emodin (B) in complex with the SSTR2 homology model; residues involved in molecular recognition are labeled while hydrogen bond between ligands and the receptor are depicted by the light blue line.*

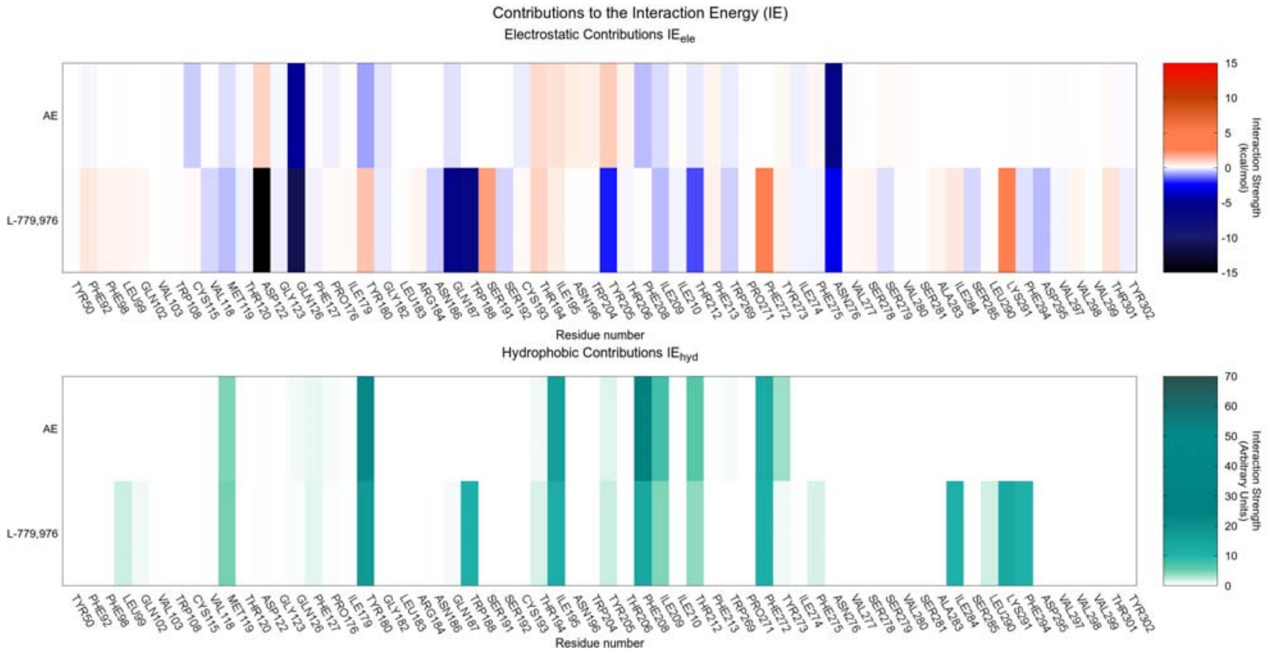

Supplementary figure 6 *Interaction energy fingerprint that summarizes, using a heat map, the electrostatic and hydrophobic contribution to binding mediated by each residue composing SSTR2 binding site with reference compound L-779,976 and AE.*

2.2. Docking studies on hSSTR5

The docking analysis of AE was repeated for the second somatostatin receptor subtype under investigation in this study, using as reference the selective non-peptidic agonist L-817,818. In supplementary figure 7 (panel A) is reported the energetically more stable complex predicted by molecular docking protocol. The portion of the orthosteric binding site occupied by the reference compound is extremely similar and in accordance with what has been described above for the SSTR2 subtype. Once again, instead, the anthraquinone compound AE displays a considerable variability of placement during docking simulation. In supplementary figure 7 (panel B) is reported a hypothesis of binding mode with the correspondent heat map analysis (supplementary figure 8).

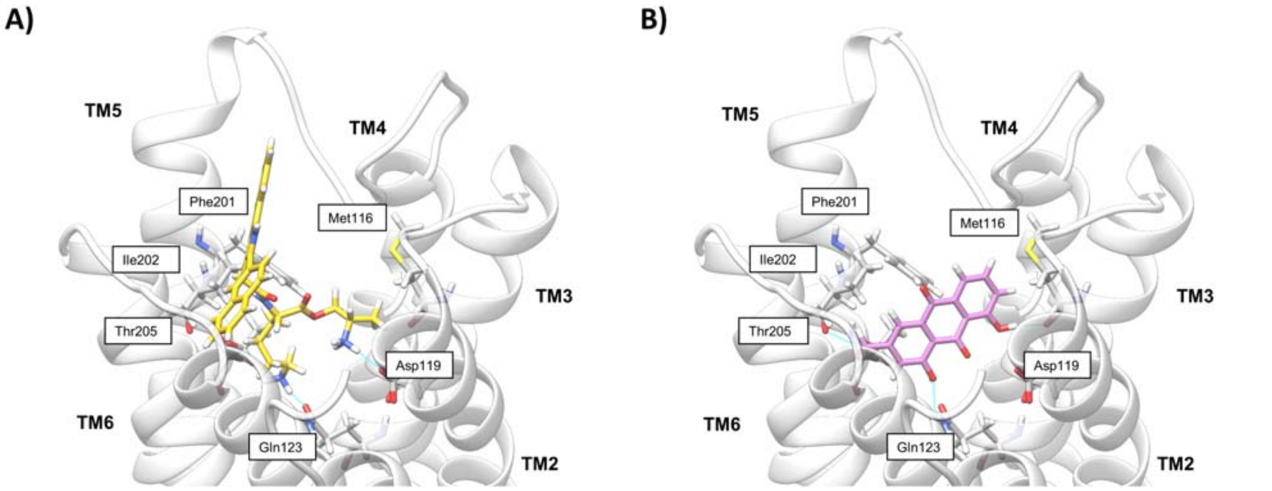

Supplementary figure 7 *Molecular docking predicted binding mode for the reference compound L-817,818 (A) and Aloe Emodin (B) in complex with the SSTR5 homology model; residues involved in molecular recognition are labeled while hydrogen bond between ligands and the receptor are depicted by the light blue line.*

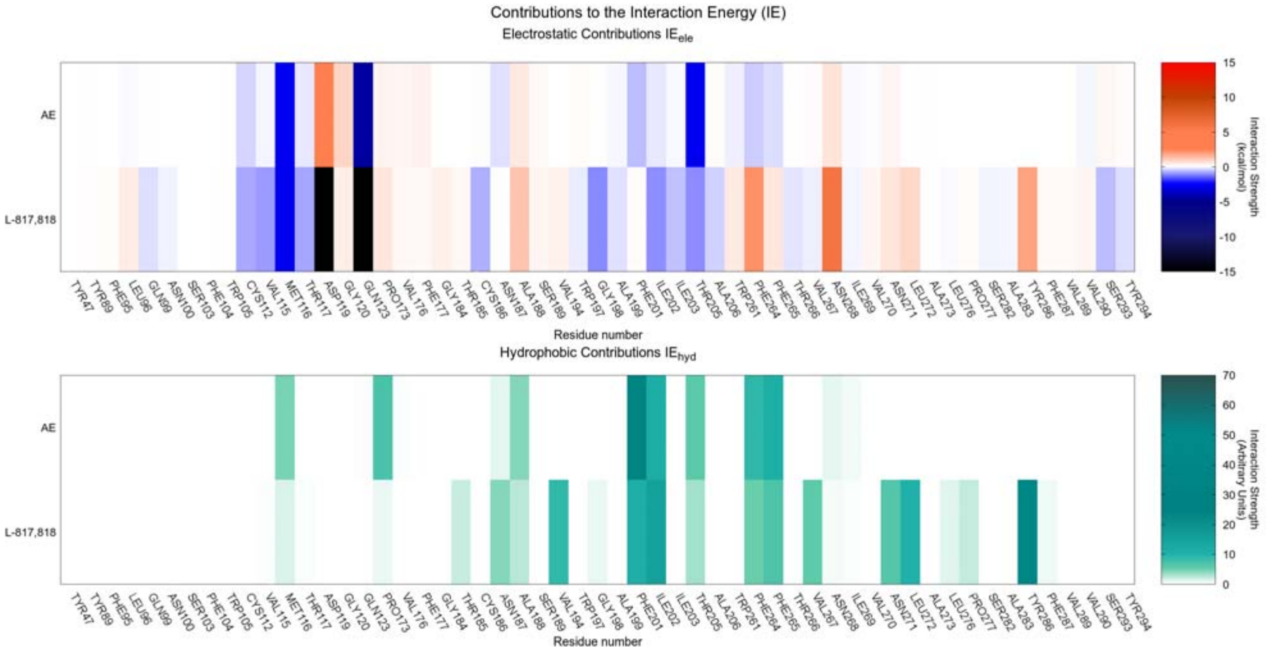

Supplementary figure 8 *Interaction energy fingerprint that summarizes, using a heat map, the electrostatic and hydrophobic contribution to binding mediated by each residue composing SSTR5 binding site with reference compound L-817,818 and AE.*

3. Discussion

A computational study was performed to investigate a possible binding mechanism between the anthraquinone compound Aloe Emodin (AE) and the human SSTRs, focusing the attention toward the subtypes 2 and 5. The docking protocol was able to predict, for both receptor subtype, a reasonable binding mode of AE, consistent with that sampled for the relatives potent and selective agonists taken as reference. From an energetic point of view, however, the interaction energy profiles have highlighted a divergence of the electrostatic and hydrophobic contribution to the AE binding, with respect to the reference compounds. These results, even if they must be considered very preliminary, allow us to hypothesize the ability of AE to recognize the somatostatin receptors. Unfortunately, It is also difficult using this computational approach, to anticipate any speculation about the potential pharmacological profile of AE toward the SSTRs, whether it acts as an agonist or an antagonist; even if, it is worthy to underline that all the compounds, currently characterized as agonists of the somatostatin receptor, present one or more positively charged groups completely absent in Aloe Emodin structure.[10]

4. References

[1] T. Che, S. Majumdar, S. A. Zaidi, P. Ondachi, J. D. McCorvy, S. Wang, P. D. Mosier, R. Uprety, E. Vardy, B. E. Krumm, et al., *Cell* **2018**, *172*, 55–67.e15.

[2] J. A. Ballesteros, H. Weinstein, *Methods Neurosci.* **1995**, *25*, 366–428.

[3] C. C. G. ULC, **2013**.

[4] H. M. Berman, J. Westbrook, Z. Feng, G. Gilliland, T. N. Bhat, H. Weissig, I. N. Shindyalov, P. E. Bourne, *Nucleic Acids Res.* **2000**, *28*, 235–242.

[5] D. A. Case, V. Babin, J. Berryman, R. M. Betz, Q. Cai, D. S. Cerutti, T. E. Cheatham Iii, T. A. Darden, R. E. Duke, H. Gohlke, **2014**.

[6] P. Labute, *Chem. Comput. Gr. Inc* **2007**.

[7] A. Waterhouse, M. Bertoni, S. Bienert, G. Studer, G. Tauriello, R. Gumienny, F. T. Heer, T. A. P. de Beer, C. Rempfer, L. Bordoli, et al., *Nucleic Acids Res.* **2018**, *46*, W296–W303.

[8] N. Guex, M. C. Peitsch, T. Schwede, *Electrophoresis* **2009**, *30*, S162–S173.

[9] G. Jones, P. Willett, R. C. Glen, A. R. Leach, R. Taylor, *J. Mol. Biol.* **1997**, *267*, 727–748.

[10] S. P. Rohrer, E. T. Birzin, R. T. Mosley, S. C. Berk, S. M. Hutchins, D. M. Shen, Y. Xiong, E. C. Hayes, R. M. Parmar, F. Foor, et al., *Science* **1998**, *282*, 737–40.

[11] J. J. P. Stewart, *J. Mol. Model.* **2007**, *13*, 1173–1213.

[12] A. Ciancetta, D. Sabbadin, S. Federico, G. Spalluto, S. Moro, *Trends Pharmacol. Sci.* **2015**, *36*, 878–890.
